# Anti-CD133 Antibody Immobilized on the Surface of Stents Enhances Endothelialization

**DOI:** 10.1155/2014/902782

**Published:** 2014-03-10

**Authors:** Jian Li, Dan Li, Feirong Gong, Shaoyan Jiang, Hua Yu, Yi An

**Affiliations:** ^1^Department of Cardiology, The Affiliated Hospital of Medical College, Qingdao University, Qingdao Shandong 266003, China; ^2^Key Laboratory for Ultrafine Materials of Ministry of Education, School of Materials Science and Engineering, East China University of Science and Technology, Shanghai 200237, China

## Abstract

Drug eluting stents successfully reduce restenosis at the cost of delayed reendothelialization. In recent years, a novel concept to enhance reendothelialization using anti-CD34 antibody coated stents which capture circulating progenitor cells (EPCs) has been developed with conflicting clinical results. CD133 is a glycoprotein expressed on circulating hematopoietic and putative endothelial-regenerating cells and may be superior to CD34 for EPCs capture stents. In the present study, anti-CD133 antibody has been successfully immobilized to the biodegradable polymeric coating material by covalent conjugation. We explore whether anti-CD133 antibody coated stents (CD133 stents) might accelerate reendothelialization in comparison with bare metal stents (BMS) through the superior ability to capture EPCs. The *in vitro* cell culture results indicate that anti-CD133 antibody functionalized polymer film significantly promotes CD133 positive cells attachment and growth compared with the unfunctionalized polymer film. In the semi-*in vivo* arteriovenous shunt model CD133 stents demonstrate much quicker specific capturing of EPCs from the blood stream than BMS within 6 hours. In a porcine coronary artery injury model CD133 stents show more effective reendothelialization in short term compared with BMS, while no significant difference in endothelial function recovery was observed between these two groups within 6-month followup.

## 1. Introduction

Implantation of drug eluting stents (DESs) has been attracting tremendous attention as positive trial results indicate their efficacy for preventing restenosis [1–3]. DESs are designed to reduce in-stent neointimal growth through the elution of cytotoxic agents that arrest the cycle of smooth muscle cell activation and proliferation [4]. Although the occurrence of in-stent restenosis has been significantly reduced, this therapy is also known to interfere with the natural vascular healing process by preventing or delaying the formation of a functional endothelial layer over the stent surface. The first-generation sirolimus and paclitaxel eluting stents are both associated with incomplete neointimal coverage [5, 6], impaired endothelial cell function [7], and improved risk of thrombosis formation [8]. Therefore, prolonged (over 6 months) dual antiplatelet therapy has been recommended in order to mitigate the risk of stent thrombosis that is associated with the incomplete healing. Based on the correlation between reendothelialization and long-term implantation results, it has been supposed that a rapid restoration of functional endothelium may provide a possible approach to improving long-term safety and efficacy of drug eluting stents [9].

Reendothelialization after vascular injury results either from local recruitment of adjacent endothelial cells [10] or from adhesion of blood-derived endothelial progenitor cells (EPCs) that differentiated and populate the surface of the stent [11]. In recent years, a novel concept to enhance reendothelialization using antibody coated stents which capture circulating progenitor cells has been raised. The EPCs capture stents (Genous Bio-Engineered R Stent, OrbusNeich, Fort Lauderdale, Florida) coated with anti-human CD34 monoclonal antibody targeted at EPCs surface antigens have been developed and shown to enhance endothelialization [12–14]. However, CD34 is not a specific marker of EPCs but rather a pluripotent stem cell marker. CD34 selected putative cells are able to differentiate into various kinds of cells including inflammatory cells and vascular smooth muscle cells, and thus only small portion (about one of 250) of the CD34 positive cells is actually EPCs [15], which may be the possible explanations for the disappointing clinical results of the Genous Stent [16, 17]. CD133, a newly discovered stem cell marker, is an earlier marker for expressing hematopoietic stem/progenitor cell (HSPCs) than CD34. In other mature blood cells, such as nucleated red blood cells, lymphocytes, myelocytic cells, mononuclear cells, and platelets, CD133 expressions were not detected. Therefore CD133 is the more specific choice for the preparation of EPCs capture stents [18].

In our previous report, copolymer of L-lactide (LLA) and 5-methyl-5-benzyloxycarbonate-1,3-dioxan-2-one (MBC) has been prepared as biocompatible and biodegradable coating materials for fabricating drug eluting stents [4, 9]. We have also proved that biomacromolecules such as heparin can be covalently immobilized to the surface of this polymer coating. Herein we explore to fabricate surface coating with endothelial cell selectivity and rapid* in situ* reendothelialization by immobilizing the anti-CD133 antibody over the stent surface. We hypothesized that the immobilization of anti-CD133 antibody enhances endothelialization and may potentially be an effective therapeutic alternative to reduce safety problems for drug eluting stents.

## 2. Materials and Methods 

### 2.1. Cells and Animals

All the cells and animal experiments were approved by the Animal Care and Use Committee of Fudan University and were in compliance with the “Guide for the Care and Use of Laboratory Animals” published by the National Academy Press (NIH Publication number 85-23, revised in 1996). Mononuclear cells (MNC) were isolated from human by the newly umbilical cord blood (HUCB) [19, 20]. HUCB samples (about 128 mL) were collected from fresh placentas with attached umbilical cords by gravity flow and divided into four equal parts for separate analysis. Heparin was used as anticoagulant. MNC were isolated by density gradient centrifugation over Biocoll (Biochrom, Berlin, Germany) for 20 min at 500 ×g and washed three times in PBS (Gibco).

CD133+ positive cells were isolated from mononuclear cells by 2-step magnetic bead purification according to the instructions of the manufacturer (Milteny Biotec). Purity was determined by fluorescence-activated cell sorter (FACS) analysis and was 92.88% ± 0.51% (*n* = 4).

Pigs (*∼*20 kg weight) were obtained from the Shanghai Animal Administration Center and received daily oral antiplatelet medication until termination.

### 2.2. Synthesis of the Polymeric Coating Material

**Figure 1:**
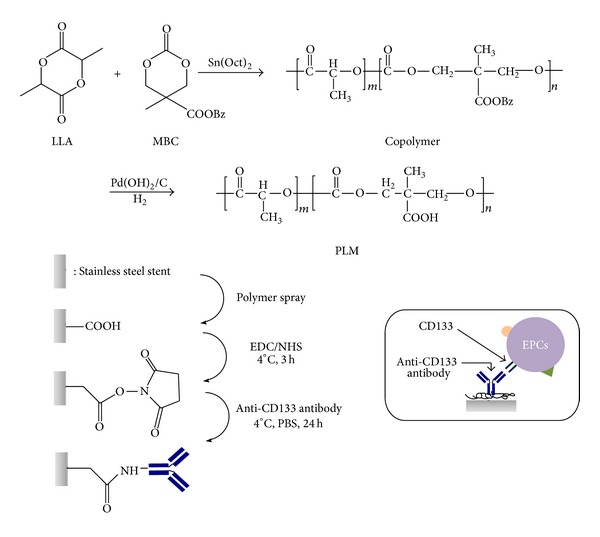
Synthesis of the polymeric coating material and preparation of the CD133 antibody immobilized stent.

Copolymer of L-lactide (LLA) and 5-methyl-5-benzyloxycarbonate-1,3-dioxan-2-one (MBC) with pendant carboxyl groups and LLA composition of 90% (PLM) was prepared according to our previous report [9]. The synthetic route of the polymeric coating material was illustrated in Figure 1. Briefly, polymerization of LLA and MBC was carried out at 110°C in vacuum for 24 h in the presence of 0.1 wt % stannous (II) 2-ethylhexanoate (Sn(Oct)_2_). Purification was carried out by dissolving the polymer in dichloromethane (DCM), which was then precipitated in excess methanol. The benzyl protective groups of the resulted copolymer were removed by the catalytic hydrogenolysis using Pd(OH)_2_/C as the catalyst. Briefly, polymer (2 g) was dissolved in 60 mL of chloroform (CHCl_3_) and then 0.6 g of Pd(OH)_2_/C (10%) in 20 mL of ethyl acetate was added into the polymer solution. After being evacuated and filled with hydrogen, the solution was stirred at room temperature for 24 h. The catalyst was then filtered and the solution was precipitated into excess methanol. After dried in vacuum at room temperature for 1 day, the polymeric coating material was obtained as a white solid.

### 2.3. Immobilization of CD133 Antibodies on the Surface of PLM Films

PLM films were prepared by dipping clean glass slides in the polymer solution in DCM and evaporating the solvent at room temperature. Then the films were immersed in 2-morpholinoethanesulfonic acid buffer (MES, Sigma-Aldrich) (0.5 M, pH 5.5) for 30 min at 4°C to allow the surface pH to equilibrate. MES buffer was then removed and the films were activated with EDC: NHS (N-ethyl-N′-(3-dimethylaminopropyl) carbodiimide, N-hydroxysuccinimide, Sigma) for 4 h at 4°C. 4 mg/mL EDC and 2.4 mg/mL NHS were used. Following activation, the samples were transferred to 0.1 mg/mL phycoerythrin-labeled CD133 antibody (CD133-PE, Milteny Biotec) in phosphate buffered saline (PBS; pH 7.6) at 4°C and kept in the dark for 24 h. CD133-PE antibody functionalized films were continuously washed with deionized water and PBS under mechanical shaking for 24 h and then for visualisation under fluorescence microscopy (Leica, German) to evaluate the presence of bound antibodies.

### 2.4. Preparation of CD133 Antibody Coated Stents (CD133 Stents)

Bare metal stents (BMS) (3.0 × 17 mm, diameter × length, Beijing Amsinomed Medical Company, China) were washed with ethanol and isopropanol and then dried in vacuum at room temperature for 1 day. Stents were weighted using a balance having 0.001 mg accuracy. PLM was accurately weighted and dissolved in HPLC grade tetrahydrofuran (THF) to prepare the coating solution. The solution was sprayed onto the surface of BMS and then dried in vacuum at room temperature for three days. The quantity of the polymeric coating material was about 50 µg for each stent or 2.78 µg/mm. After surface equilibrated with MES buffer, the polymer coated stents were activated with EDC and NHS and then CD133 antibody (Milteny Biotec) was covalently immobilized on the surface of the stents which was similar to the process of CD133-PE antibody immobilization on the polymeric film surfaces. The process was illustrated in Figure 1.

### 2.5. *In Vitro* Cell Study

CD133+ progenitor cells attachment and proliferation were performed by seeding the cells in Dulbecco's modified Eagle's medium (DMEM, Gibco) in 35-mm glass culture dishes coated with PLM and CD133 antibody functionalized PLM at a density of 5 × 10^5^/cm^2^. Next, 3 mL of medium supplemented with 150 ng of vascular endothelial growth factor (VEGF, Santa Cruz) was added to the dishes and mixed with the cells. The cells were then cultured in a humidified incubator equilibrated with 5% CO_2_–95% air for 14 days. After 3-day culture nonadherent cells were removed and fresh medium with 50 ng/mL of VEGF was added. The medium was replaced every three days. The morphology of the cells attached was examined using phase contrast light microscopy every day and microphotographs were taken at days 3, 7, and 14. At day 14, the cells were also stained with Dil-labeled acetylated LDL (Dil-ac-LDL, Biomedical Tech.) and observed under fluorescence microscopy to evaluate the proliferation assay.

### 2.6. Arteriovenous Shunt Model

The arteriovenous shunt model was employed for the comparison of EPCs capture ability from the blood stream of CD133 stents and BMS. Under sterile conditions, both femoral arteries and veins of the pig were isolated and side branches were clipped and cut. A disinfected 3 × 30 cm (diameter × length) mm extracorporeal circulation pipe with valve, wherein 3 BMS and 3 CD133 stents were placed and expanded, was connected as a shunt system to complete the circuit between the arterial and venous circulation. After 1 hour and 6 hours, the pipe was cut open and the stents were taken out and carefully washed with PBS. Cells attached on the surface of the stents were identified by Dil-ac-LDL and Hoechst 33342 (Sigma) double staining. All the animals survived in this experiment.

### 2.7. Stent Implantation

On the procedure day, fourteen pigs were anesthetized with ketamine (20 mg/kg intramuscularly) and xylazine (2 mg/kg intramuscularly). BMS (*n* = 14, 5 for one week, 4 for two weeks, and 5 for four weeks) and CD133 stents (*n* = 16, 6 for one week, 5 for two weeks, and 5 for four weeks) were implanted in 2-3 coronary arteries per pig by random assignment to anatomic location. The resulting stent-to-artery ratio was about 1.2–1.3 : 1 by quantitative coronary angiography analysis. The animals were anesthetized with ketamine (20 mg/kg) and xylazine (2 mg/kg) for follow-up angiography in the same orthogonal views before death with 20 mL of potassium chloride intracoronary injection. Then the stented arteries were carefully dissected from the myocardium and cut into two pieces, each about 9 mm long for cross-sections preparation and SEM imaging. In this experiment all the animals survived until termination.

### 2.8. Endothelialization of the Stented Arteries and Evaluation of Endothelium Function

Endothelialization of stented arteries was examined using SEM 1 week, 2 weeks, and 4 weeks after stent implantation. Endothelium function after stent implantation was estimated by measuring the coronary vasomotor reactivity in response to acetylcholine (Ach, 60 mg, performed at an infusion rate of 1 mL/min) infusion within 6-month followup [4]. Five pigs receiving two different stents each and ten stents (5 BMS and 5 CD133 stents) were used in this experiment. End diastolic images for each segment were chosen and analyzed with the automated edge detection program (FD-10, Philips, Best, Netherland). Two orthogonal views with less foreshortening or without overlapping of side branches were selected and averaged for biplane assessment by two experts blinded to stent type. About 5 mm distal to the site of stenting was chosen for analysis. Changes in coronary diameter in response to Ach coronary infusion were expressed as percent changes versus baseline angiograms.

### 2.9. Statistical Analysis

Numerical data are presented as mean ± standard error of the mean. Each measurement was repeated independently three times. Continuous variables were compared by one-way ANOVA in Origin 7.0 (Microcal, USA) and a value of *P* ≤ 0.05 was considered as a significant difference.

## 3. Results and Discussion

### 3.1. Synthesis of the Polymeric Coating Material and Preparation of CD133 Stents

**Figure 2:**
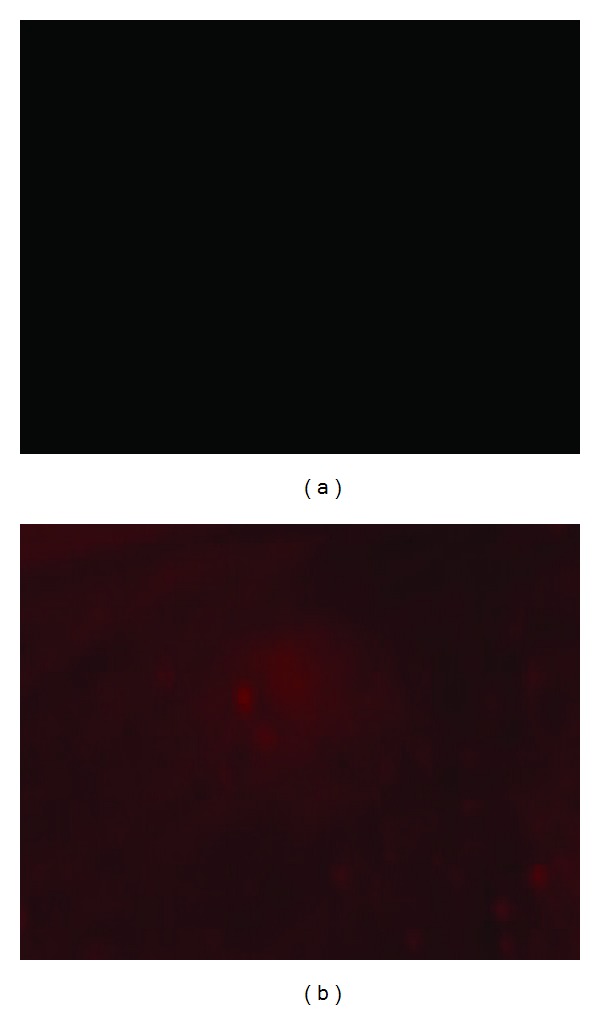
Fluorescence images of PLM film (a) and PE-labeled CD133 antibody immobilized PLM film (b).

In our previous report [9], we have proved that biomacromolecules such as heparin can be covalently immobilized to the surface of PLM films. The significant loading of carboxyl groups on the surface of the films allows for subsequent biomolecules conjugation such as antibodies and peptides. The fluorescence images of PLM film and PE-labeled CD133 antibody functionalized PLM film were illustrated in Figure 2. There was no red fluorescence that can be observed on the PLM films, while, in the case of CD133-PE conjugated PLM film, fluorescence tagging of the CD133 antibodies showed uniform distribution on the surface of the polymer film under fluorescence microscopy, indicating the antibodies have been successfully conjugated to the carboxyl groups on the surface of the polymer film. The films were continuously washed with PBS under mechanical shaking for 24 h before measurements, with no observed reduction in the mean fluorescence intensity measured, showing antibodies were stably bound.

### 3.2. *In Vitro* Cell Study

**Figure 3:**
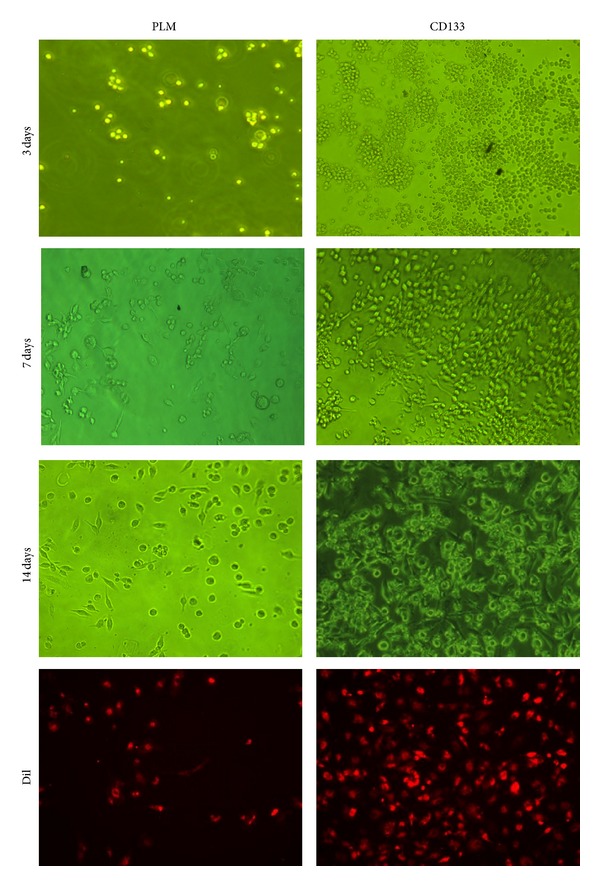
Phase contrast microscopic images of EPCs attachment and proliferation on PLM films and CD133 antibody immobilized PLM films within 14-day culture. Fluorescence images of attached EPCs stained with Dil-ac-LDL demonstrate improved adhesion and spread-out morphology on CD133 antibody immobilized films compared to the unmodified PLM films within 14-day culture.

Preliminary studies were performed to evaluate* in vitro* CD133+ selected putative EPCs attachment and proliferation on CD133 antibody functionalized PLM films and the unfunctionalized PLM films for different time intervals (3, 7, and 14 days).  Figure 3 showed the morphologies of cells adhered to and proliferated on these two surfaces within 14 days after seeding. The seeding density of our EPCs in the medium on the polymer surface was 5 × 10^5^ cells/cm^2^. Our results indicated that at this seeding density seeding of EPCs on PLM films resulted in poor cellular adhesion at day 3, with limited proliferation by day 7. Addition of CD133 antibodies allowed significantly improved cellular adhesion and proliferation on the surfaces of PLM films. The number of attached EPCs 3 days after seeding increased evidently on the surfaces of CD133 antibody functionalized PLM films when compared to the unmodified surfaces. At this time point, most of the cells adhered to these two kinds of polymer surfaces presented a spherical shape and were uniformly distributed. Up to day 7 of culture, a part of the cells began to be flat and polygonal in shape and were better spread out. At day 14, a lot of cells on the surfaces of PLM films still presented a spherical shape, while most of the cells on the CD133 antibody functionalized PLM surfaces achieved a more mature conformation with a spindle-like shape, which was also identified by the proliferation results studied with fluorescein Dil-ac-LDL staining (red).

### 3.3. Arteriovenous Shunt Model

**Figure 4:**
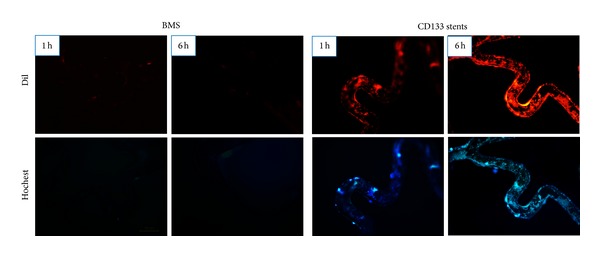
Comparison of the capability to capture EPCs in the arteriovenous shunt model between BMS and CD133 stents for 1 hour and 6 hours. Samples were rinsed with PBS and stained with Dil-ac-LDL and hochest.

Fluorescence images of BMS and CD133 stents flushed in the arteriovenous shunt model for 1 hour and 6 hours after being stained with Dil-ac-LDL and hochest were shown in Figure 4. Both groups of stents were carefully taken out and rinsed with PBS. In the arteriovenous shunt model, when installed in the blood circulation system up to 6 h, BMS shows no red color fluorescence on their surfaces under fluorescent microscopy, indicating no endothelial cells or endothelial progenitor cells attached on BMS surfaces within this time period. It was, however, totally different in the case of CD133 stents. The number of cells which were captured by CD133 stents in the blood circulation system was much higher than BMS. By the first hour it was statistically estimated to be more than 30% of the surfaces of CD133 stents covered by red color fluorescence, indicating the CD133 stents can effectively capture endothelial cells or blood-derived endothelial progenitor cells from the bloodstream. At this time point, most cells were still round, indicating that endothelial progenitor cells circulating in the host bloodstream were possibly the main source for these newly adhered cells [21]. After 6 hours, more than 50% of the CD133 stents surfaces were covered by red fluorescence. The experimental results showed that CD133 antibody coating specifically captured EPCs in the peripheral blood and the captured EPCs can be differentiated into endothelial cells by the induction of VEGF and accelerate the reendothelialization process in the coronary stent system.

### 3.4. Morphological Evaluation of the Stented Arteries

**Figure 5:**
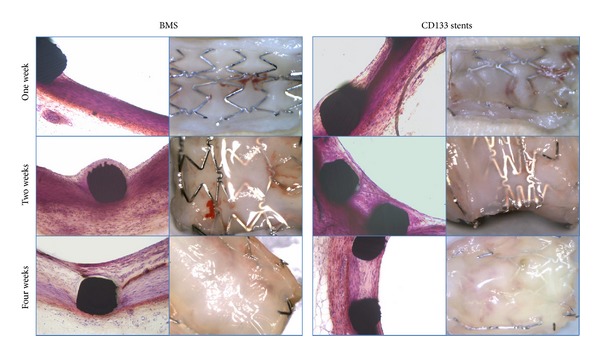
Typical microphotographs of the stented blood vessel and high-power micrographs of the inner wall of the blood vessels in normal porcine coronary arteries 1 week, 2 weeks, and 4 weeks after stent implantation. Left: BMS; right: CD133 stents.

After implantation for one week, two weeks, and four weeks, the stented arteries were harvested, cross-section-sliced, and H-E-stained. The micrographs of the inner wall of the blood vessels were also taken. No significant neointimal hyperplasia was observed in either BMS or CD133 stents within 4 weeks of implantation. Also no obvious inflammation responses were found in either of the groups. Typical optical microscopic photographs of the stented arteries and the inner wall of the blood vessels after stent implantation are shown in Figure 5. In the first two weeks, BMS and CD133 stents had significant different appearance with fibrin-platelet deposition and neointima formation. At the first week of implantation, most of the struts of BMS were still bare in the blood stream and some BMS surfaces were covered with fibrous tissues and blood cell networks. In the case of most CD133 stents, after only one week, a thin and smooth layer of neointima with no coagulations or excrescences formed on the stent surface. The percent area stenosis for BMS and CD133 stent at this time point was 6.2 ± 3.6% and 10.4 ± 3.7%, respectively, and the difference was significant (*P* < 0.05). In the second week, neointimal layers could be found on the surface of some BMS samples, while, on the surface of CD133 stents, the neointimal tissues seemed to be more integrated. The difference was not significant in the percentage of stenosis for BMS and CD133 stents at this time point (*P* > 0.05, 10.8 ± 6.4% versus 12.3 ± 5.2%). After 4 weeks of implantation, the healing processes on all BMS and CD133 stents were found to be completed and no significant difference in percentage of stenosis was observed (*P* > 0.05).

### 3.5. Endothelialization of the Stented Arteries and Evaluation of Endothelium Function

**Figure 6:**
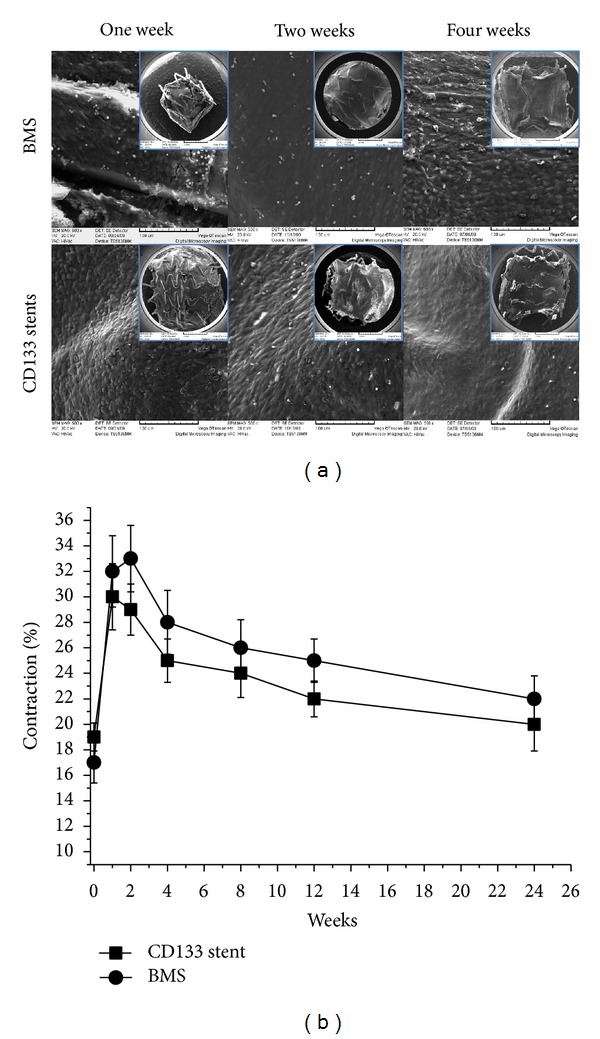
Typical SEM images (magnification: ×500, ×25 for insets) of the inner wall of stented arteries 1 week, 2 weeks, and 4 weeks after stent implantation (a) and relaxation responses of coronary segments distal to stents within 24-week followup (b).

Endothelial recovery is an essential component for vascular healing by providing critical structural and antithrombogenic functions [22]. The progress of reendothelialization of stented arteries was examined using SEM at 1 week, 2 weeks, and 4 weeks after stent implantation as shown in Figure 6(a). Both the amount and the morphology of the endothelial cells on the stent surfaces were analyzed. At the first week, almost no endothelial cells attached to the BMS surface could be observed, but a thin layer of fibrin-platelet deposition and acute inflammatory cells aggregation could be found, while the CD133 stents were covered by endothelial cells which were randomly and tightly arrayed. Most cells were still round, indicating that endothelial progenitor cells circulating in the host bloodstream were possibly the main source for these cells. For the second week of development, endothelial cell attachment could be found to occur on the BMS surface with a relative low density. The endothelialization of the BMS surfaces was statistically estimated to be around 30% as observed by SEM whereas the CD133 stents reached almost 100%. The cells on the BMS surface appeared to be infantile and undeveloped, while on the CD133 stents the luminal surface and the stent struts had been covered with confluent shuttle-like endothelial cells. They formed a continuous mat which was aligned with the direction of the flow of blood. After 4 weeks, there was no significant difference in endothelialization for BMS and CD133 stents; complete endothelialization with a cobblestone structure covering the smooth muscle layer could be observed in both BMS and CD133 stents treated arteries. The only difference was that the cell arrays were more pronounced in the case of the CD133 stents.

It is well known that coronary stenting leads to disruption of the endothelial layer and leaves a thrombogenic metallic surface exposed to the blood stream [23]. Therefore, endothelium function evaluation was prospectively designed to compare coronary endothelial dysfunction between the arteries treated with BMS and CD133 stents as well as morphology observation. The CD133 stents could obviously accelerate the endothelial cell restoration from the SEM images, while, in this study, although there was a trend that the segments distal to the BMS be more strongly constricted to the Ach infusion within 6-month followup (Figure 6(b)), the difference was not significant (*P* > 0.05). The results indicated that rapid restoration of endothelial cells on the stent surface does not mean rapid endothelium function recovery. So it is still unclear whether the rapid endothelialization of CD133 stents will be associated with any long-term clinical benefits in comparison with traditional drug eluting stents. The truly healing process after stent implantation may take much longer time than the period of endothelial cell restoration on the stent surface. In the HEALING IIB trial to evaluate the bioengineered CD34 antibody coated stent (Genous Stent), an overall 3% incidence of definite in-stent thrombosis rate was observed, which was not better than the sirolimus and paclitaxel eluting stents [24]. On the other hand, the EPCs capture stent technology did not sufficiently impede clinical restenosis rates and late luminal loss at 6-month angiographic followup [24]. So more detailed studies about the efficacy of CD133 stents need to be designed and proposed.

## 4. Conclusions

In conclusion, these studies demonstrated that immobilization of anti-CD133 antibody on the stent surface can enhance endothelial cells coverage and may potentially be an effective therapeutic alternative to improve currently available drug eluting stents.

## References

[1] Sousa JE, Costa MA, Sousa AGMR (2003). Two-year angiographic and intravascular ultrasound follow-up after implantation of sirolimus-eluting stents in human coronary arteries. *Circulation*.

[2] Stone GW, Ellis SG, Cox DA (2004). A polymer-based, paclitaxel-eluting stent in patients with coronary artery disease. *The New England Journal of Medicine*.

[3] Serruvs PW, Morice M-C, Kappetein AP (2009). Percutaneous coronary intervention versus coronary-artery bypass grafting for severe coronary artery disease. *The New England Journal of Medicine*.

[4] Shen L, Gong FR, Tian WJ (2013). Anti-inammatory effect of arenic trioide eluting stent in a porcine coronary model. *BioMed Research International*.

[5] Kotani J-I, Awata M, Nanto S (2006). Incomplete neointimal coverage of sirolimus-eluting stents. Angioscopic findings. *Journal of the American College of Cardiology*.

[6] Wilson GJ, Nakazawa G, Schwartz RS (2009). Comparison of inflammatory response after implantation of sirolimus- and paclitaxel-eluting stents in porcine coronary arteries. *Circulation*.

[7] Hofma SH, van der Giessen WJ, van Dalen BM (2006). Indication of long-term endothelial dysfunction after sirolimus-eluting stent implantation. *European Heart Journal*.

[8] Finn AV, Joner M, Nakazawa G (2007). Pathological correlates of late drug-eluting stent thrombosis: strut coverage as a marker of endothelialization. *Circulation*.

[9] Gong F, Cheng X, Wang S, Zhao Y, Gao Y, Cai H (2010). Heparin-immobilized polymers as non-inflammatory and non-thrombogenic coating materials for arsenic trioxide eluting stents. *Acta Biomaterialia*.

[10] Robinson KA, Roubin G, King S, Siegel R, Rodgers G, Apkarian RP (1989). Correlated microscopic observations of arterial responses to intravascular stenting. *Scanning Microscopy*.

[11] Banerjee S, Brilakis E, Zhang S (2006). Endothelial progenitor cell mobilization after percutaneous coronary intervention. *Atherosclerosis*.

[12] Shirota T, Yasui H, Shimokawa H, Matsuda T (2003). Fabrication of endothelial progenitor cell (EPC)-seeded intravascular stent devices and in vitro endothelialization on hybrid vascular tissue. *Biomaterials*.

[13] Co M, Tay E, Lee CH (2008). Use of endothelial progenitor cell capture stent (Genous Bio-Engineered R Stent) during primary percutaneous coronary intervention in acute myocardial infarction: intermediate- to long-term clinical follow-up. *The American Heart Journal*.

[14] Aoki J, Serruys PW, van Beusekom H (2005). Endothelial progenitor cell capture by stents coated with antibody against CD34: the HEALING-FIM (Healthy Endothelial Accelerated Lining Inhibits Neointimal Growth-First in Man) registry. *Journal of the American College of Cardiology*.

[15] Larsen K, Cheng C, Tempel D (2012). Capture of circulatory endothelial progenitor cells and accelerated re-endothelialization of a bio-engineered stent in human ex vivo shunt and rabbit denudation model. *European Heart Journal*.

[16] Beijk MAM, Klomp M, Verouden NJW (2010). Genous endothelial progenitor cell capturing stent vs. the Taxus Liberté stent in patients with de novo coronary lesions with a high-risk of coronary restenosis: a randomized, single-centre, pilot study. *European Heart Journal*.

[17] Beijk MAM, Klomp M, Van Geloven N (2011). Two-year follow-up of the genous endothelial progenitor cell capturing stent versus the taxus libert stent in patients with de Novo coronary artery lesions with a high-risk of restenosis: a randomized, single-center, pilot study. *Catheterization and Cardiovascular Interventions*.

[18] Sedaghat A, Sinning JM, Paul K (2013). First in vitro and in vivo results of an anti-human CD133-antibody coated coronary stent in the porcine model. *Clinical Research in Cardiology*.

[19] Al-Azemi TF, Bisht KS (1999). Novel functional polycarbonate by lipase-catalyzed ring-opening polymerization of 5-methyl-5-benzyloxycarbonyl-l,3-dioxan-2-one. *Macromolecules*.

[20] Juliane E, Stefanie K, Gergely J (2003). Endothelial progenitor cell culture and differentiation in vitro: a methodological comparison using human umbilical cord blood. *Cardiovascular Research*.

[21] Meng S, Liu Z, Shen L (2009). The effect of a layer-by-layer chitosan-heparin coating on the endothelialization and coagulation properties of a coronary stent system. *Biomaterials*.

[22] Thorin E (2001). Influence of nitric oxide synthase inhibition and endothelin-1 receptor blockade on acetylcholine-induced coronary artery contraction in vitro in dilated and ischemic cardiomyopathies. *Journal of Cardiovascular Pharmacology*.

[23] Kim JW, Seo HS, Park JH (2009). A prospective, randomized, 6-month comparison of the coronary vasomotor response associated with a zotarolimus versus a sirolimus-eluting stent. Differential recovery of coronary endothelial dysfunction. *Journal of the American College of Cardiology*.

[24] den Dekker WK, Houtgraaf JH, Onuma Y (2011). Final results of the HEALING IIB trial to evaluate a bio-engineered CD34 antibody coated stent (GenousStent) designed to promote vascular healing by capture of circulating endothelial progenitor cells in CAD patients. *Atherosclerosis*.

